# Imagining How Lines Were Drawn: The Appreciation of Calligraphy and the Facilitative Factor Based on the Viewer’s Rating and Heart Rate

**DOI:** 10.3389/fnhum.2021.654610

**Published:** 2021-06-30

**Authors:** Kazuki Matsumoto, Takeshi Okada

**Affiliations:** Department of Educational Psychology, The University of Tokyo, Tokyo, Japan

**Keywords:** graphonomics, art viewing, empirical aesthetics, heart rate, smartphone-based PPG, recognition of the process of creation, paralanguage, human communication

## Abstract

For this study, we examined how recognizing the writing process of calligraphy influences the cognitive and affective processes related to appreciating it, with the aim of contributing to both graphonomics and the psychology of aesthetics. To this end, we conducted two Web-based experiments in which some participants were instructed to view calligraphy by tracing it with their eyes (the tracing method), while others were told to feel free to think and imagine whatever they wanted. Study 1 (*N* = 103) revealed that the tracing method elicits stronger admiration, inspiration, and empathy in viewers. Study 2 (*N* = 87) showed that the tracing method decreases the average heart rate of those who do not frequently engage in calligraphy appreciation as they gaze at calligraphy for a minute-and-a-half (during the second half of the stimulus duration); this suggests that the tracing method could keep viewers from becoming bored while looking at calligraphy. In sum, the tracing method has positive effects on viewing calligraphy. From a broader perspective, the results imply that how in detail viewers recognize the process of creating an artwork will be a key determinant of art appreciation. In addition, our findings demonstrate how we can measure cardiac activities using the emerging technology of the photoplethysmogram (PPG).

## Introduction

The way people communicate with each other is one of the most important research topics in human science, and is tied to many disciplines including psychology, neuroscience, anthropology, sociology, linguistics, information science, and evolutionary biology (cf. Shannon, 1948; Wiley, 1983; Craig, 1999; Littlejohn and Foss, 2010). Although calligraphy is not the most frequently used form of human communication, as we will see below, studying calligraphy is valuable. It contributes to clarifying written, non-verbal, or artistic communication, areas of communication that remain largely unexplored. In this introduction, we must first see how calligraphy can be positioned within these communication subfields and how it shares certain characteristics with other types of communication.

It is common to distinguish between verbal and non-verbal communication. Needless to say, verbal communication is crucial in social activities, and people have valued it since ancient times; this is reflected in the fact that many kinds of verbal activities have been refined and made more sophisticated from generation to generation, eventually becoming so-called “art” forms like literature or rhetoric.

How about non-verbal communication? Verbal communication is inseparable from non-verbal communication (cf. Jones and LeBaron, 2002; Hall et al., 2019); if we think about paralinguistic phenomena, we can readily grasp the connection. Paralanguage is normally defined as vocal behavior accompanied by aspects of words (such as pitch and volume), or, more broadly, the aggregation of “vocal, kinesics (gestural), and proxemics (spatial) channels” (Loveday, 1982; cf. Pennycook, 1985; Hall et al., 2019). These definitions connote that any linguistic communication inevitably contains paralinguistic features. Further, paralanguage plays a substantial role in relaying information such as a speaker’s affective state (e.g., Scherer et al., 1973; Johnstone and Scherer, 2000; Scherer, 2003) or intention (e.g., Hellbernd and Sammler, 2016); it is far from a mere peripheral occurrence. Paralinguistic information can be an important part of some artistic activities in the same way that verbal information is. Singing is a good example. When we listen to someone singing, the lyrics convey verbal messages, but we usually pay more attention to how (or in what tone) they are sung, which is the counterpart of paralanguage in everyday face-to-face conversations (for the similarity between everyday vocal expression and musical performance (see Juslin, 2013; Juslin and Laukka, 2003).

Whereas “paralinguistic” research only centers on phenomena in vocal communication or textual simulations, such as emoticon (Luangrath et al., 2017), written linguistic elements are seldom studied as a kind of paralanguage (for exceptions, see Kilyeni, 2009 and Oshiki et al., 2010). However, many examples show that people receive, as well as frequently and actively gather, information from “paralinguistic” components of written communication, in the sense that linguistic content (what is written) always accompanies visible characteristics (how something is written). This influences how readers form impressions. For instance, many organizations in countries such as France and the United States use techniques from graphology for personnel selection (King and Koehler, 2000). Among the aristocracy of pre-modern Japan, the quality of handwriting was seen as fundamental to spousal selection, in addition to literature skills (Gatten, 1986). As for the art of calligraphy, it can be found in virtually any culture with letters. In terms of handwriting, “paralanguage” is too crucial to numerous human behaviors to be left unexamined. If we can psychologically clarify how we receive “paralinguistic” information in written communication, we can make a significant contribution to the entire field of communication research. Based on the above points, we experimentally examined how handwritten objects mediate social interactions from the perspective of the perceiver or viewer, with a focus on Japanese calligraphy. In the remaining part of this introduction, we review the literature on graphonomics and the psychology of aesthetics, argue how our study theoretically contributes to both fields, and illustrate the purpose of the experiments.

### Graphology and Graphonomics

Historically, people in literate societies have been interested in the individuality of handwriting. Yang Xiong, an ancient Chinese philosopher, stated, “The spoken is voice of spirit. The written is picture of spirit” (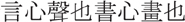). This idea is also familiar in the contemporary West, with many methods of assessing personality through handwriting systematized as graphology. However, although graphology has a long history and is widely popular, most scientific research to date has failed to support its validity (Simner and Goffin, 2003). In the first half of the 20^th^ century, some psychologists perceived graphology as a pseudoscience, similar to phrenology or palmistry (Allport and Vernon, 1933); this critical stance has become more broadly accepted – but not dismissed – in the field of psychology.

Unlike graphology, graphonomics is a more recent and empirical discipline; it refers to the “scientific and technological effort involved in identifying relationships between the planning and generation of handwriting and drawing movements, the resulting spatial traces of writing and drawing instruments (either conventional or electronic), and the dynamic features of these traces” (van Gemmert and Teulings, 2004). As implied in the above definition, existing graphonomic research focuses on written or drawn traces, or the within-individual processes in which they are produced. Notwithstanding, words are written for communication in the first place; thus, we cannot understand entire systems of writing behavior if we ignore what handwriting (including “paralinguistic” features) expresses to readers. Given that a writer might (perhaps unconsciously) modify her/his handwriting so that it looks good to readers (based on her/his own reading experience) during the stage of movement planning or right in the middle of writing, even in a situation where the intrapersonal handwriting process is the object of research interest, it will only be partially revealed, without discussing how readers perceive handwriting. Hence, it is vital for graphonomic research to explore the cognitive processes that underlie perceiving someone’s handwriting.

For this study, we considered an aesthetically valued style of handwriting; that is, calligraphy. There are two advantages to this approach. First, calligraphy is written with the pursuit of an ideal visual appearance of characters (both for the calligrapher and the viewer), and has rich implications for studying people’s handwriting preferences. The process of forming a preference for another person’s characters is likely rooted in the same basis as the process of developing a goal when writing. This signifies that clarifying the mental processes of aesthetic impression formation in viewing calligraphy is meaningful for graphonomics, in the sense that we can investigate the higher-order cognitive processes underlying general writing behavior (such as planning how to make letters look better). Second, in exploring calligraphy as art, we can be informed by theories in the psychology of aesthetics for a deep discussion. Grounded in the theories in question, we expect that calligraphy works convey some information to the viewer other than semantic content, which is not limited to speculative calligraphers’ personalities graphologists have suggested so far, but may include recognition of calligraphers’ skills, or of the process of writing calligraphy (Matsumoto and Okada, 2019), as described in the next section.

As we review related studies in the psychology of aesthetics in the following section, we should keep in mind that many of them focus on visual arts, not verbal arts. In the context of the psychology of aesthetics, while diverse investigations deal with visual and literal arts, virtually no research has covered calligraphic works. Since we want to shed light on non-verbal or “paralinguistic” functions in visual features of calligraphy (rather than purely verbal ones), we expect theories of visual art to have useful implications, to which we primarily refer.

### Art-Viewing and Viewers’ Recognition of the Process of Creating Artworks

When viewing calligraphy, what determines our evaluation, and what kinds of cognitive and affective processes are involved? Findings from the psychology of aesthetics provide a framework for addressing this question. Recently, numerous studies in this area have shown that the mental process by which viewers integrate an artwork’s physical features with their own memories or knowledge (whether consciously or not) is essential in establishing their impressions. Leder et al. (2004) information processing model contains five sequential stages in individual art appreciation: (1) perceptual analysis, (2) implicit memory integration, (3) explicit classification, (4) cognitive mastering, and (5) evaluation. This model continues to be updated. Today, a lot of researchers agree that the “evaluation” stage – after “cognitive mastering” in the original model – is not necessarily located at the end of the actual art-viewing process. That is, during relatively long-term viewing, reconsideration and re-evaluation of artwork can be observed; hence, we can expect impressions of art to change as time goes on (Pelowski and Akiba, 2011; Pelowski et al., 2017, 2020).

While the information processing model is concerned with classifying and segmenting components of art appreciation in terms of cognitive psychology, another approach gaining interest centers on viewers’ internal representations, generated through a series of processing stages. Following the influential work of Tinio (2013), art creation and viewing are in a symmetrical relationship in which viewers mentally trace artists’ process of creation in reverse order. Bullot and Reber’s (2013) psycho-historical framework also suggests that art can convey causal-historical information, although without emphasis on the processing order underscored by Tinio’s (2013) mirror model. These frameworks are similar in that a communicative aspect of art creation and viewing is elucidated. From the standpoint of communication theory, various empirical studies have shown that how a viewer (i.e., the “receiver” in the communication model) evaluates an artwork (receiving the “message” from the “signal”) depends on her/his knowledge of its creator (the “transmitter”) or the process of creation, as in the typical communication model, where reception of a message depends on the encoding/decoding rules. For example, Jucker et al. (2014) demonstrated that how lay people define an object as art (or not) and how they like it is affected by artist-related instructions given prior to viewing, such as regarding whether the artist intentionally created the work.

Whereas many studies have revealed the effect of directly presented information about the creator upon the viewer’s evaluation, viewers can adopt a new way of viewing art following a change in their own cognitive structure, even without such a direct presentation of information as the one used by Jucker et al. (2014; Matsumoto and Okada, 2019). By comparing viewers with and without prior experience of creating origami works in a laboratory, Matsumoto and Okada (2019) found that viewers’ own creative experiences enabled them to discern and imagine the process of creating artworks (creative origami works) by others in more detail, followed by the promotion of the aesthetic experience in a positive direction, including admiration elicited by upward social comparison. Matsumoto and Okada (2019) discovered that individual differences in cognitive processes of art-viewing – which are especially salient in the comparison between experts and novices (cf. Leder et al., 2004; Leder et al., 2012; Bullot and Reber, 2013) – are reproducible to some degree with novices’ acquired experiences of creation. On the other hand, whether the key determinant of the aesthetic experience is the creative experience, or the way in which the process of creating a work is perceived, remains unresolved; Matsumoto and Okada (2019) imply the latter based on post-hoc correlational analysis.

Considering the above, for the current study, we utilized another approach to establish whether how we perceive the process of creating artworks can influence the cognitive process of appreciation, including the overall evaluation. More specifically, we investigated whether the simple cognitive orientation of “how to look at artwork” – without requiring any special equipment or training – can change one’s impression of art in diverse ways. Not only did we examine causal relationships that prior research did not fully test; we also indicated the generalizability of the finding in Matsumoto and Okada (2019) due to adopting different types of works (compared to our previous study), which is considered a contribution to the psychology of aesthetics. We had to choose materials suitable for our purpose, and Chinese/Japanese calligraphic works sufficed (mentioned next), as well as graphonomic interest (described previously).

### Chinese/Japanese Calligraphy and the “Tracing Method”

Chinese calligraphy dates back to the beginning of the use of Chinese characters (more than 3,000 years ago at least). Since Japanese calligraphy is a branch of Chinese calligraphy and has the same historical origins, they are quite similar. While the most striking difference is in the characters used (*kana* are sometimes used along with Chinese characters in Japanese calligraphy), as for aspects relevant to the current study (such as the process of creation or standards of the value of works), they are very similar, since Chinese traditions (including calligraphy) have been regarded as role models by Japanese intellectuals, for whom writing calligraphy has been possible throughout almost all of Japanese history (considering this similarity, hereafter, we will not distinguish between them and will refer to both styles as “calligraphy” unless otherwise noted). Unlike modern Western art, traditional calligraphy is not necessarily intended to be exhibited in public. In addition, conventionally speaking, calligraphy is not a fully independent genre, but is inextricably related to diverse forms of written communication. Thus, people today perceive copies of poems or sutras, and personal writings such as letters or diaries, as valuable forms of calligraphy.

In virtue of using calligraphy as experimental material, we were able to effectively investigate what role the viewer’s recognition of the process of creating artworks plays in art-viewing, which is an important research topic in the psychology of aesthetics (as mentioned earlier). The reason for this is that the cognitive orientation regarding the recognition of the writing process is attainable by giving a relatively simple instruction on perceptual performance, even when a non-expert contemplates calligraphy. This is because although calligraphy has a static form (like painting), it more directly and vividly presents traces of creation on paper to viewers compared to other forms of visual art, who can refer to the rules of character stroke order and can imagine how (and in which order) lines were drawn if they scrutinized the visual features of lines (such as a blur or gradation). Moreover, our experiments benefitted from the fact that Japanese elementary and junior high schools offer a class on penmanship; almost all Japanese people have had the experience of writing imitatively in a way similar to traditional calligraphy. Due to these circumstances, the Japanese participants likely had a cognitive foundation for recognizing the calligrapher’s writing process, without any additional creative experiences. As such, by guiding viewers’ perceptual process with the textual instructions, such as “Please pay attention to how lines were drawn,” we were able to experimentally manipulate their recognition of the process of creating artworks in a different way from that of Matsumoto and Okada (2019), thereby providing new evidence for the importance of that kind of recognition in art-viewing.

In addition to stressing that viewers should pay attention to the process of writing while appreciating calligraphy, much of the literature recommends specific methods for viewing. In particular, imitative writing of the same characters in a work of calligraphy (whether with an ink brush or by tracing them with one’s finger) is considered effective (e.g., Ishikawa, 2011; Shi, 2020). Below, we will discuss how this technique can facilitate the beginner’s detailed recognition of the calligrapher’s writing process. Under normal reading conditions, letters or characters are processed quickly enough on average for 4 to 5 letters to be covered by a single eye fixation when reading English sentences (Samuels et al., 2010), where it is neither necessary nor common to pay close attention to the fine features of letters or characters. This is considered true for Japanese to some extent, which transmits a comparable amount of information per fixation versus English and German (Fukuda and Fukuda, 2009). This is also the case in situations where novices are exposed to calligraphy. Even if they are told to be “aware” of the process of writing, they might still construct limited mental representations based on the relatively small amount of information available from their accustomed habit of reading. In contrast, by spending longer time for imitative writing or tracing characters with one’s finger, the viewer can pay close attention to the physical features that convey rich information about how the brush was used – which may also be related to the mental aspect of the calligrapher’s writing process (cf. Matsumoto and Okada, 2019) – and thereby think about them at a deeper level (see Okada and Ishibashi, 2017 for a similar discussion in the context of art creation).

Although using a brush or finger is usually recommended for appreciating calligraphy, the previous paragraph implies that we can derive similar benefits by moving only our eyes, as if tracing each stroke, without any other body movement. If employing this method while contemplating calligraphy, viewers are expected to construct a detailed recognition of the writing process based on the work’s paralinguistic features. Otherwise – especially without any cognitive orientation for recognition of the writing process through an instruction – novice viewers are likely to compress visual information into a rather simple impression, such as “strong” or “delicate.” For the present study, based on the discussion in this section, we used Japanese calligraphy as experimental material and manipulated an instruction (the independent variable) between participants in terms of how to view calligraphy. We expected this to determine the direct factor in changing their impressions; that is, the cognitive processes involved in recognizing the writing process. In order to influence recognition of the writing process so as to promote their impressions of calligraphy, we instructed the participants to move their eyes as if tracing the characters (hereafter called the “tracing method”) under one condition.

### The Current Study

Through experiments using Japanese calligraphy, we explored how recognizing the process of creating artworks may affect the cognitive and affective processes of viewing them. Unlike Matsumoto and Okada (2019), we directly manipulated the viewer’s perception through an instruction instead of a creative experience. More specifically, we compared two conditions within this paradigm: one scenario involved a group of participants whom we instructed to use the tracing method; the participants whom we subjected to the second condition were not given any cognitive orientation. Further, to more accurately identify pertinent factors, we added a third condition in the first experiment: We instructed this group of participants to pay attention to the writing process of calligraphy without using the tracing method. By doing so, we were able to establish whether novice participants could construct detailed mental representations of the writing process, so as to update their impressions via a single instruction, without any specific method or procedure (such as the tracing method).

Although we primarily employed the same measurements as Matsumoto and Okada (2019) for the sake of theoretical continuity (namely liking and admiration as aesthetic impressions), we also introduced two new measurements. First, we gauged the degree to which the participants felt inspired by calligraphy using a slightly modified version of a questionnaire developed by Ishiguro and Okada (2015), which was originally based on Thrash and Elliot’s (2003) Inspiration Scale (see Supplementary Table 1 for details). According to Ishiguro and Okada (2020), inspiration – which mediates between art-viewing and creative behavior – is related to the social comparative processes, and can be encouraged by the viewer’s “dual focus”; that is, attention paid to both others (e.g., “His approach was to take numerous photos”) and self (e.g., “I only took a few shots to obtain the best photo”). Based on Smith’s (2000) discussion of inspiration and other social comparative emotions – as a crucial element of the theoretical foundation of both Ishiguro and Okada (2020) and Matsumoto and Okada (2019) – inspiration is fairly likely to be promoted when the recognition of the process of creating artworks changes enough to elicit admiration. This is because inspiration is akin to admiration in Smith’s (2000) classification. Both emotions are “upward” and “assimilative”; the only essential difference is whether they are “dual” focused or “other” focused. Thus, when admiration is elicited, a large part of the basis for inspiration is already satisfied. If the duration of viewing is not too brief, viewers’ attention may shift to themselves or others from time to time, and inspiration and admiration may co-occur when contemplating a single work. Therefore, we measured how viewers were inspired by calligraphy and expected that when admiration was encouraged, inspiration would be also fostered.

Second, we used a physiological indicator: heart rate value estimation by photoplethysmogram (PPG). PPG is a non-invasive optical method for gauging the relative changes in blood volume in an area of tissue with blood capillaries on the skin surface (such as a finger or earlobe); it can be used to detect heartbeat or pulse and has been increasingly adopted in recent years (Gil et al., 2010; Kamshilin et al., 2015; Lohani et al., 2019). Cardiovascular activity is a commonly employed physiological parameter in the psychology of aesthetics due to its ease of use (e.g., Libby et al., 1973; Nell, 1988; Tschacher et al., 2012). Many studies have explored the link between autonomic nervous system activity (e.g., heartbeats) and affective feelings (mediated by brain regions such as the anterior cingulate cortex, Critchley et al., 2013; see Kreibig, 2010 for a review). Among affective states, boredom is tied to art-viewing and is associated with heart rate; that is, people tend to exhibit higher heart rates when bored (London et al., 1972; Merrifield and Danckert, 2014; Raffaelli et al., 2018).

Recent research suggests that PPG can be obtained via commercial smartphones without any specialized equipment (Kurylyak et al., 2012; Garcia-Agundez et al., 2017; Guede-Fernández et al., 2020). Smartphone-based PPG allows us to conduct experiments remotely, which is especially valuable in the recent situation of COVID-19 pandemic. This technique has not yet been perfected and is rarely introduced, particularly in psychology. Thus, our findings offer useful insights for practical application. One thing to note here is that smartphone-based PPG in a remote experiment is likely to increase the cognitive load on participants and to make experiments complicated. To address this issue, we conducted two experiments. We designed the first one to be simple; we only included psychological rating scales as dependent variables. The second experiment contained all measurements.

We posited that the tracing method would have a positive impact on participants’ admiration and inspiration based on the discussion so far. Related to that, it was possible for the average heart rate to differ between conditions, influenced by changes in affective states elicited by the instructions. In addition, we examined effects on other psychological variables: “the degree of empathy for the calligrapher” (empathy) and “the degree to which imagination is triggered” without any specific hypothesis.

## Study 1

### Method

#### Participants

A total of 103 participants took part via their own Web-connected computers instead of in a laboratory. We recruited them through a crowdsourcing service and paid them each 1,000 JPY for completing the tasks, which took less than an hour. Only native adult speakers of Japanese with no visual impairments were allowed to participate.

#### Stimuli

**FIGURE 1:**
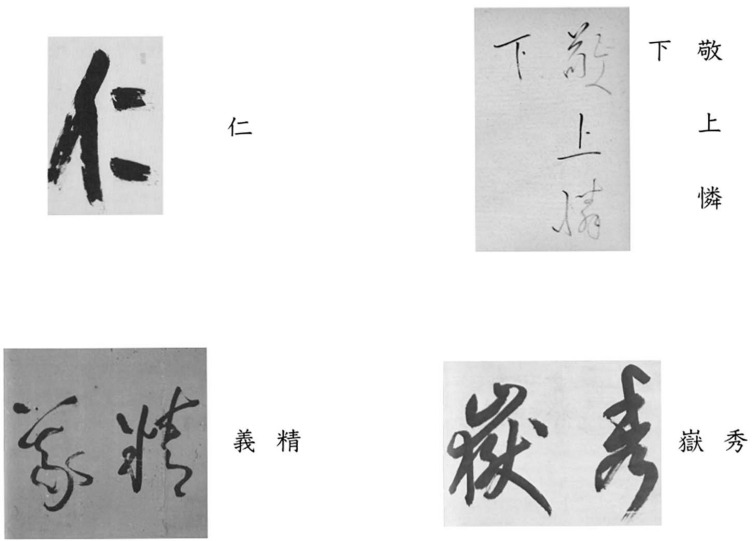
The four calligraphy works used as stimuli (with printed font).

We chose four works of Japanese calligraphy (Figure 1) based on the following criteria: (a) they are regarded as classic, established works; (b) the phrases are brief, and contain characters that are easy to read; and (c) they do not differ too much from modern styles, and are considered readable for beginners in calligraphy. In the end, each image only had Chinese characters, the number of which ranged from 1 to 4 (see Supplementary Table 2 for detailed information about each work).

#### Experimental Conditions

We randomly assigned the participants to one of three conditions: the orientation with the tracing method (the “Tracing Group”; *n* = 34); the orientation without the tracing method (the “No-Tracing group”; *n* = 34); and the non-orientation (the “Control Group”; *n* = 35). For the Tracing Group, we asked the participants to view the calligraphy by tracing each line in the order in which it was supposed to have been drawn, and by imagining both the mental and physical processes of writing. As for the meaning of “tracing,” we only told them to move their eyes; there was no mention about hands or fingers. We instructed the participants in the No-Tracing Group in the same way as the Tracing Group, except for the part about the “tracing method.” We told them to imagine the process of creating artworks while viewing it without describing any specific method. For the Control Group, we did not give the participants any orientation and told them to feel free to think about and imagine whatever they wanted. Also, for all participants, there were explicit statements that any kind of thought or imagination that is not suggested in our instruction is not prohibited at all (see Supplementary Table 3 for detail).

#### Procedure

**FIGURE 2:**
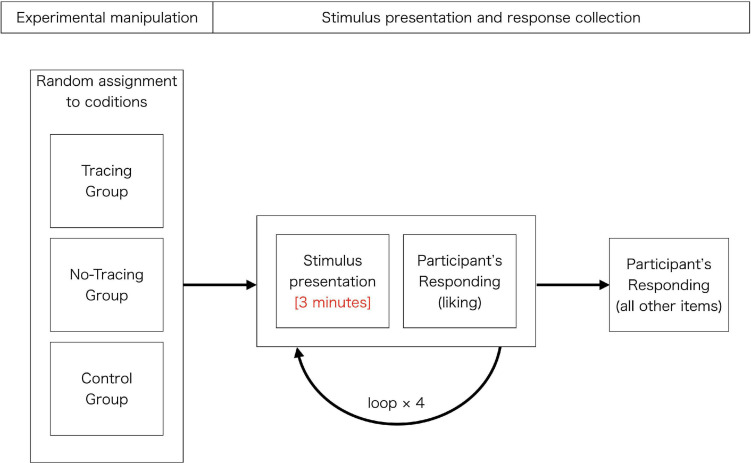
The procedure of experiment in Study 1.

After reading a broad description of the tasks (see Figure 2 for a schematic representation) in an online document, each participant provided informed consent and began the tasks using their favored browser, accessing our web server. We required them to complete the tasks using a computer with a stable connection to a network in a quiet, non-distracting environment. In order to let them behave as naturally as possible, we did not strictly control their physical or software-related conditions.

After the participants adjusted their settings, they read a text that suggested how they should view the calligraphy (the manipulation on this was described in the previous section and Supplementary Table 3). The experimental stimuli were then presented sequentially for 3 min each. With the aim of enabling participants to read older calligraphy stress-free, each stimulus consisted of a set of three images for a single artwork: (1) calligraphy only; (2) calligraphy with the same characters using printed font; and (3) only a short description of the meaning of the written word and information about the author’s name, as well as the author’s year of birth and death. These images were displayed one by one; the participants were allowed to switch between the three images at any time by pressing certain keys. Following each stimulus presentation, they were asked to answer two 7-point Likert items measuring the degree to which they liked each presented work, and the degree to which they liked meaning of the written word in each work. After all the stimuli were shown once, they were asked to answer the other Likert items: admiration; empathy; imagination; recognition of the process of creation (awareness of each mental and physical aspect); inspiration; filler items which were not included in any analysis but contributed to avoiding participants being overly aware of what indicators we theoretically focused on (such as “I couldn’t see what it said”); and measurement of their personal characteristics (including the frequency they view calligraphy and length of time spent learning calligraphy). Because of the assumed high correlation between the two personal variables, only the frequency of viewing (with its interaction with the condition) was used as an individual difference factor in every analysis in Study 1. Except for some cases such as inspiration, these items consisted of simple statements such as “I felt admiration” and scales of agreement ranging from 0 (do not agree at all) to 6 (very strongly agree) and they were asked for each stimulus and thus repeated as many times as the number of stimuli, namely four times (see Figure 2 and Supplementary Table 1). There was no time limit for any item. We implemented the main program with jsPsych (de Leeuw, 2015), a JavaScript library for creating behavioral experiments in a Web browser.

### Results and Discussion

For each variable measured more than once, we calculated each participant’s score by averaging the stimuli in each session, which, in turn, we used for statistical analysis as a data point. Unless otherwise stated, we compared each dependent variable between groups in analysis of covariance (ANCOVA) models with Tukey’s correction (the “glht” function of the package “multcomp” of R; Hothorn et al., 2008). We included a dummy variable for the condition of instruction, the score of the frequency of viewing calligraphy, and the interaction term of both variables as predictor variables. Following a recommendation from the existing literature on multiple comparison (Hsu, 1996; Wilkinson, 1999), we did not consider rejecting the global null-hypothesis as a prerequisite for pairwise comparisons; nor did we test the global null-hypothesis beforehand in those models, since we were interested in identifying the differences between each of the two groups in those cases.

#### Validation of Assignments and Experimental Manipulation

To check whether the participants were appropriately assigned in terms of their attitudes toward calligraphy, we calculated the mean and standard deviations (SD) of their scores for the frequency of viewing calligraphy for each condition (see Table 1). As one-way analysis of variance (ANOVA) revealed no significant differences among them, *F*(2, 100) = 0.03, *p* = 0.966, there is no evidence to suggest that the assignments were biased regarding the frequency of viewing calligraphy.

**TABLE 1:** Descriptive statistics for all rating values in Study 1.

Variable	Tracing group	No-tracing group	Control group
Frequency of viewing	0.41 (0.74)	0.41 (0.78)	0.37 (0.69)
Awareness of physical creation	4.66 (0.87)	4.07 (1.02)	3.33 (1.16)
Awareness of mental creation	4.16 (1.16)	4.42 (0.94)	3.36 (1.36)
Admiration	3.58 (1.05)	3.15 (1.18)	2.94 (1.40)
Liking of a work	3.35 (1.12)	3.12 (1.01)	3.04 (1.16)
Liking of meaning of word	4.15 (0.84)	4.04 (0.78)	4.01 (0.82)
Inspiration	3.51 (1.38)	3.38 (1.24)	2.87 (1.67)
Empathy	3.43 (0.98)	3.30 (1.03)	2.90 (1.23)
Imagination	3.85 (1.23)	4.05 (0.97)	3.42 (1.27)

*Standard deviations appear in parentheses.*

Further, to verify whether the manipulation of the instructions influenced the participants’ cognitive processes as expected, we calculated the mean and SD of their scores for (a) the degree to which they were aware of the physical process of writing calligraphy (awareness of physical creation) and (b) the extent to which they were aware of the mental process of writing calligraphy (awareness of mental creation; see Table 1). We expected that participants in the Tracing Group and No-Tracing Group would have higher scores than the Control Group for both (a) and (b). As for (a) awareness of physical creation, there was a significant difference between the Tracing Group and the Control Group, *b* = 1.48, *t*(97) = 5.20, *p* < 0.001, and also between the No-Tracing Group and the Control Group, *b* = 0.89, *t*(97) = 3.14, *p* = 0.006. We did not detect any significant differences between the Tracing Group and the No-Tracing Group, *b* = 0.59, *t*(97) = 2.07, *p* = 0.101. Multiple regression analysis indicated no other significant effect for the frequency of viewing and the interactions (Table 2). As for (b) awareness of mental creation, like the former results, there was a significant difference between the Tracing Group and the Control Group, *b* = 1.00, *t*(97) = 3.15, *p* = 0.006, and also between the No-Tracing Group and the Control Group, *b* = 1.18, *t*(97) = 3.75, *p* < 0.001. We did not witness any significant differences between the Tracing Group and the No-Tracing Group, *b* = −0.18, *t*(97) = −0.57, *p* = 0.835. In addition, multiple regression analysis only showed a significant effect for the frequency of viewing (*p* = 0.031, Table 2) other than the above. These findings were consistent with our prediction. Hence, we considered the participants’ cognitive processes to be appropriately orientated.

**TABLE 2:** Multiple regression analyses in Study 1.

Dependent variable	Predictor		*b*	*t*	*p*
Awareness of physical creation					
	Intercept		3.17***	15.99	<0.001
	Condition (with the baseline of control group)				
		Tracing group	1.48***	5.20	(<0.001)
		No-tracing group	0.89**	3.14	(0.006)
	Frequency of viewing		0.42	1.62	0.108
	Interaction (condition × frequency)				
		Tracing condition	−0.39	−1.11	0.271
		No-tracing condition	−0.40	−1.16	0.247
Awareness of mental creation					
	Intercept		2.13***	14.11	<0.001
	Condition (with the baseline of control group)				
		Tracing group	1.00**	3.15	(0.006)
		No-tracing group	1.18***	3.75	(<0.001)
	Frequency of viewing		0.63*	2.19	0.031
	Interaction (condition × frequency)				
		Tracing condition	−0.53	−1.35	0.181
		No-tracing condition	−0.35	−0.90	0.368
Admiration					
	Intercept		2.75***	12.06	<0.001
	Condition (with the baseline of control group)				
		Tracing group	0.79*	2.42	(0.045)
		No-tracing group	0.13	0.40	(0.917)
	Frequency of viewing		0.50	1.70	0.093
	Interaction (condition × frequency)				
		Tracing condition	−0.40	−0.98	0.328
		No-tracing condition	0.15	0.38	0.701
Liking of a work					
	Intercept		2.91***	13.82	<0.001
	Condition (with the baseline of control group)				
		Tracing group	0.51	0.30	(0.212)
		No-tracing group	0.06	0.30	(0.980)
	Frequency of viewing		0.37	1.35	0.179
	Interaction (condition × frequency)				
		Tracing condition	−0.55	−1.43	0.155
		No-tracing condition	0.01	0.02	0.982
Inspiration					
	Intercept		2.53***	9.54	<0.001
	Condition (with the baseline of control group)				
		Tracing group	0.93*	2.46	(0.041)
		No-tracing group	0.55	1.47	(0.309)
	Frequency of viewing		0.92**	2.68	0.009
	Interaction (condition × frequency)				
		Tracing condition	−0.82	−1.75	0.084
		No-tracing condition	−0.20	−0.44	0.659
Empathy					
	Intercept		2.72***	13.32	<0.001
	Condition (with the baseline of control group)				
		Tracing group	0.70*	2.40	(0.048)
		No-tracing group	0.38	1.32	(0.388)
	Frequency of viewing		0.50	1.89	0.062
	Interaction (condition × frequency)				
		Tracing condition	−0.47	−1.30	0.198
		No-tracing condition	−0.00	−0.01	0.995
Imagination					
	Intercept		3.20***	14.40	<0.001
	Condition (with the baseline of control group)				
		Tracing group	0.67	2.11	(0.093)
		No-tracing group	0.74	2.33	(0.056)
	Frequency of viewing		0.59*	2.05	0.043
	Interaction (condition × frequency)				
		Tracing condition	−0.64	−1.62	0.108
		No-tracing condition	−0.32	−0.82	0.415

Parenthesized values were corrected for multiple comparisons.

*p < 0.05; **p < 0.01; ***p < 0.001.

#### Aesthetic Impression

**FIGURE 3:**
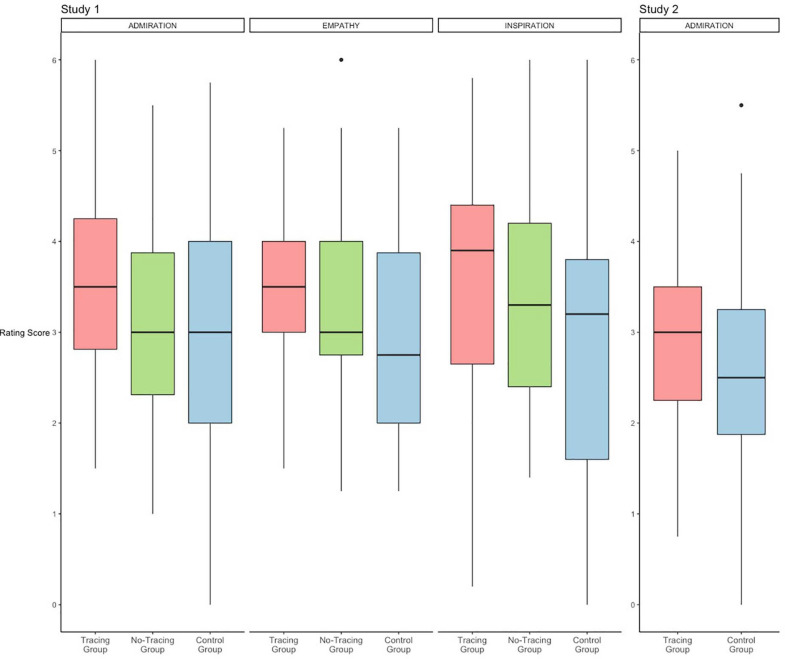
Boxplots of rating scores in Study 1 and 2. This does not include dependent variables in which significant effects were not found.

We calculated the mean and SD of participants’ scores for admiration of works of calligraphy (Table 1 and Figure 3). Tukey pairwise comparisons indicated a significant difference only between the Tracing Group and the Control Group, *b* = 0.79, *t*(97) = 2.42, *p* = 0.045, and no significant differences between the other pairs; *b* = 0.66, *t*(97) = 2.02, *p* = 0.114 (tracing–no-tracing), *b* = 0.13, *t*(97) = 0.40, *p* = 0.917 (no-tracing–control). Multiple regression analysis demonstrated no other significant effect for the frequency of viewing and the interactions (Table 2). Likewise, we computed the mean and SD of the participants’ scores for liking works of calligraphy (Table 1). For this analysis, we omitted one participant due to missing data. As opposed to the results of admiration, Tukey pairwise comparisons signaled no significant differences between any two groups; *b* = 0.45, *t*(96) = 1.50, *p* = 0.295 (tracing–no-tracing), *b* = 0.51, *t*(96) = 1.70, *p* = 0.212 (tracing–control), *b* = 0.06, *t*(96) = 0.19, *p* = 0.980 (no-tracing–control). Multiple regression analysis revealed no other significant effect for the frequency of viewing and the interactions (Table 2).

The former outcome is in line with our hypothesis based on Matsumoto and Okada (2019), and supports the notion that the degree of admiration we have for artworks depends on how we recognize the process of creating them. On the other hand, the latter result does not support the idea that liking art depends highly on one’s recognition of the process of creation. Since Matsumoto and Okada (2019) showed that whether viewers have had creative experiences significantly affects both their admiration and liking, it may seem contradictory at first glance that our results are different for admiration and liking. However, given the differences between the characteristics of admiration and liking, they can be interpreted in a way consistent with our previous study. Admiration is a type of social comparative emotion, and that is seen as the reason why our recognition of the process of creation affects it. According to Matsumoto and Okada (2019), when viewers compare themselves to outstanding others in terms of recognizing others’ processes of creation in a detailed way, they should feel intense admiration, as they become confident of the difficulty or “unachievability” of creation by others. In contrast, liking, a typical measurement of aesthetic judgment by viewers, is formed through more varied mental processes (cf. Leder et al., 2004). For example, people may become attached to a painting because it depicts a scene they like, or because it was drawn by a beloved child, rather than due to its outstanding achievement. Therefore, although liking and admiration are likely to correlate, since liking is determined by factors other than how we recognize the writing process, the impact of the instruction seems to be diluted.

For additional analysis, for each condition, we calculated the correlation coefficient between liking of the work itself and liking of meaning of the word in it. The Pearson’s *r* was 0.68, 0.64, and 0.70 for the Tracing Group, the No-Tracing Group, and the Control Group, respectively (all *p* < 0.001). Since the degree of liking the meanings of words are formed quite independently of the recognition of the writing process, our results align with the perspective that other factors determine the overall extent to which one likes a work of calligraphy.

Regarding the effect of instruction in the No-Tracing Group, there were no obvious results, with no significant differences compared to either the Tracing Group or the Control Group. Although our findings do not directly support the existence of a clear difference between the Tracing Group and the No-Tracing Group, the fact that the difference of the Control Group only occurred in the Tracing Group suggests the importance of the perceptual intervention of the tracing method, which should lead to a detailed recognition of the writing process (as described in the Introduction). Because this discussion can be applied to any other dependent variable, hereafter, we will omit the same kind of discussion related to the No-Tracing Group, unless there is a noteworthy outcome.

#### Inspiration

We calculated the mean and SD of the participants’ scores for inspiration by viewing calligraphy (Cronbach’s alpha coefficient was 0.93; Table 1 and Figure 3). Tukey pairwise comparisons only revealed a significant difference between the Tracing Group and the Control Group, *b* = 0.93, *t*(97) = 2.46, *p* = 0.041. We did not find any significant differences between the other pairs; *b* = 0.38, *t*(97) = 0.99, *p* = 0.583 (tracing–no-tracing), *b* = 0.55, *t*(97) = 1.47, *p* = 0.309 (no-tracing–control). In addition, multiple regression analysis only showed a significant effect for the frequency of viewing (*p* = 0.008, Table 2), other than the above.

These results are in line with our hypothesis based on the dual focus model of inspiration (Ishiguro and Okada, 2020), which suggests that a comparative process with attention paid to both others and oneself leads to inspirational experiences. Further, we found that the frequency of viewing calligraphy correlates with the intensity of inspiration, which is consistent with Ishiguro and Okada (2019), who showed that art experience (including the frequency of art appreciation) correlates with inspiration.

#### Other Measurements

We calculated the mean and SD of participants’ scores for empathy toward calligraphers (Table 1 and Figure 3). Tukey pairwise comparisons only indicated a significant difference between the Tracing Group and the Control Group, *b* = 0.70, *t*(97) = 2.40, *p* = 0.048, but no significant differences between the other pairs; *b* = 0.32, *t*(97) = 1.08, *p* = 0.527 (tracing–no-tracing), *b* = 0.38, *t*(97) = 1.32, *p* = 0.388 (no-tracing–control). Multiple regression analysis revealed no other significant effect for the frequency of viewing and the interactions (Table 2).

These outcomes imply that the tracing method encourages viewers to empathize with calligraphers more than when no orientation is given. This seems to be mediated by reinforcement of the viewer’s “dual focus” on self and others’ writing process in the Tracing Group, which is also considered as a cause of inspiration. Given that the dual focus process (particularly the mental aspect of creation) inevitably involves assuming the artist’s perspective, it is natural that the dual focus would foster empathy, which is closely associated with perspective-taking (Healey and Grossman, 2018).

We also calculated the mean and SD of the participants’ scores for imagination (Table 1). Tukey pairwise comparisons showed no significant difference between any pair; *b* = 0.67, *t*(97) = 2.11, *p* = 0.093 (tracing–control), *b* = −0.06, *t*(97) = −0.20, *p* = 0.978 (tracing–no-tracing), *b* = 0.74, *t*(97) = 2.33, *p* = 0.056 (no-tracing–control). Multiple regression analysis only indicated a significant effect for the frequency of viewing (*p* = 0.043, Table 2).

Although these outcomes do not support the idea that differences in instruction and cognitive orientations influence the degree to which a viewer’s imagination is triggered, since the differences in both pairs of tracing–control and no-tracing–control approach significance, it would be difficult to conclude their independence solely from those outcomes. In addition, we found that people who appreciate calligraphy frequently are more likely to have richer imaginations. As imagination is one of the typical ways to enjoy art, this correlation is very natural.

## Study 2

In Study 2, retaining the same hypotheses as Study 1, we added the heart rate analysis using smartphone-based PPG.

### Method

#### Participants

A total of 81 participants took part through their own Web-connected computers instead of in a laboratory. We recruited them through a crowdsourcing service or via recruitment statements on social networking sites (SNS) and paid them 1,000 JPY (for participants from the crowdsourcing service) or a 1,500 JPY electronic gift certificate (for participants from the SNS) for completing the tasks, which took about an hour. Since physiological measures are sensitive to gender and age, we limited the participants to males between the ages of 18 and 39 (*M* = 25.00, SD = 2.50). In addition, all participants were native speakers of Japanese and had no visual or cardiac impairments. We only excluded one participant in advance from all analyses because of overly extreme responses for the psychological Likert items (only reporting either end of the range of the Likert scale for all items).

#### Stimuli

We chose the same four works of Japanese calligraphy as in Study 1 (Figure 1).

#### Experimental Conditions

While there were three conditions in Study 1, we excluded the No-Tracing Group from Study 2 because we did not find any major differences between the No-Tracing and the Control Group. Hence, we had the remaining two conditions: the Tracing Group and the Control Group. The instructions for each condition were the same as those used in Study 1; that is, we told participants in the Tracing Group to view calligraphy by tracing each line and imagining the writing process, and we told those in the Control Group to think and imagine whatever they wanted, without any orientation.

There was some difference in the sample size of each condition, despite random assignment (*n* = 37 in the Tracing Group; *n* = 43 in the Control Group). Further, this was intertwined with the place from where they were recruited (crowdsourcing service: *n*_t_ = 8, *n*_c_ = 18; SNS: *n*_t_ = 29, *n*_c_ = 25; the “*t*” or “*c*” suffix indicates the “Tracing Group” or the “Control Group”). This difference was caused by the discrepancy between conditions for number of participants who had agreed to participate in the experiment, but did not actually finish (not counted in the values above). Since we did not detect such cases in Study 1, the procedures for measuring the PPG in Study 2 may have been complicated beyond their expectations so as to lead to this situation. In order to partial out these effects, in the regression analyses, we included a dummy variable for the place of recruitment as a covariate (see the Results and discussion section for details).

#### Procedure

**FIGURE 4:**
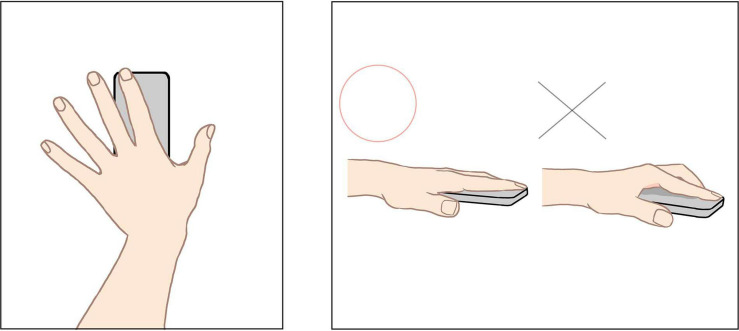
Examples of images for the instruction of measuring PPG with smartphone used in the experiment in Study 2. The left part represents left hand on smartphone with the index finger covering the camera lens, seen from above. The right part represents good and bad example of measuring seen from the side, as the left one is favorable because the entire finger is in close contact with the smartphone while otherwise in the right one the index finger may get tired and shake, causing noise in the video.

The procedure as a whole was very similar to that of Study1. The participants read a broad description of the tasks, provided informed consent, and started the Web-based experiment, which included instructions for calligraphy, the stimulus presentation, and completing the Likert items. The only important difference from Study 1 was that for Study 2, the participants were required to use their own smartphones with cameras to record a video of the skin of the tip of their left index finger. Specifically, for the first task of the experiment, we asked them to first free up space on their device’s memory to record a video, and then asked them to record their skin for 30 s as a trial using a diagram (Figure 4 depicts some examples). Upon finishing the trial recording, they uploaded the video file to a server specified by the experimenter without pause. The experimenter checked the uploaded file for any flaws in the video as soon as possible. If there was no problem, he sent a message to continue the experiment; otherwise, he requested that the participant re-take the video until there were no more issues with it (the first recording needed to have been completed correctly, because it was also used as a baseline in the following analysis). Subsequently, the participants read a text suggesting how they should view calligraphy (depending on the conditions), in the same way as Study 1. From this point, the sequence of the experiment returned to the same form as in Study 1. When the experimental stimuli were presented sequentially for 3 min after that, the participants recorded their fingers over the entire period of stimulus presentation, stopping and re-taking the recording each time. Since the participants had to keep their left hand on the camera lens of their smartphones, they used their right hand to operate the keyboard to switch between images. We synchronized the timing of data acquisition between stimuli presentation and PPG recordings using electronic sounds from each participant’s computer (which ran the program for the experiment); the sounds were heard at the beginning and end of each stimulus presentation and recorded in the video of the skin.

Because there is a circadian rhythm of heart rate (e.g., Massin et al., 2000) – which should be controlled for the sake of data quality – we limited the time that participants could start the experiment to between 13:00 and 18:00. All other parts of the procedure were the same as in Study 1.

#### Analysis of PPG

**FIGURE 5:**
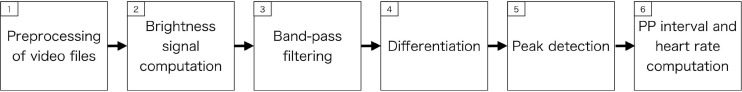
The main processing blocks of the heart rate estimation.

**FIGURE 6:**
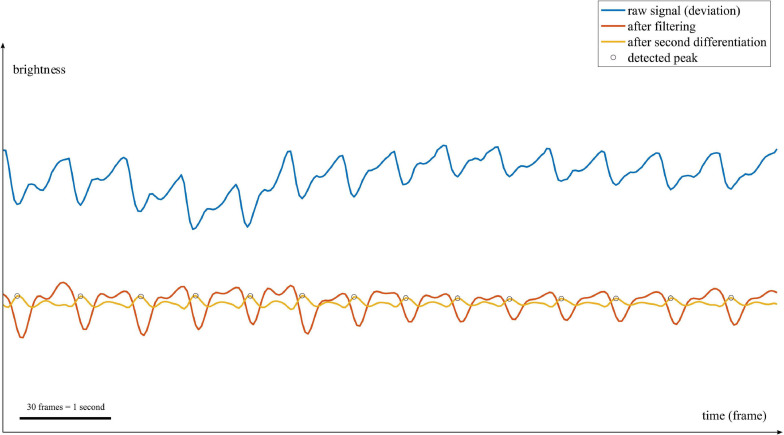
An example of PPG waveforms. The horizontal axis represents time (the sequence of frames in a video file), and the vertical axis represents image brightness reflecting blood flow. The length of the black horizontal bar at the bottom left of the figure corresponds to 30 frames (=1 s). The blue line shows the raw signal in the second block of Figure 5 (each value of brightness on the vertical axis is a deviation from the mean value, that is, the value from which we subtracted its mean value). The red line shows the bandpass-filtered signal in the third block. The yellow line shows the second derivative signal in the fourth block. The black circles show every detected peaks in the fifth block.

Figure 5 portrays the processing for estimating the average heart rate. In each block, the process was as follows: (1) We first preprocessed all video files submitted by the participants to convert them (the videos were originally shot on different smartphone models and using different frame rate settings) to 30 frames per second. At the same time, we identified the frame numbers in each video where the electronic sounds, emanating from the experimental program, were recorded. We then used the frame numbers to trim the videos. (2) For every frame, we calculated the mean value of all pixels of red intensity in RGB, regarding it as an index of “brightness” at each time point. The change in brightness over time is thought to reflect the change in blood flow. (3) We band-pass filtered the obtained signal over 0.67-3.83Hz (≈ 40-230 beats per minute) using a second-order Butterworth filter to reduce noise. (4) We differentiated the waveform of brightness twice to make the peak derived from the heartbeat more prominent (Figure 6 displays this contrast; see Guede-Fernández et al., 2020 for an example of using differentiation). (5) We detected the peaks using the “findpeaks” function in MATLAB. (6) Based on the detected peaks, we calculated the pulse-to-pulse intervals (PPI). Because there were often problems with skipping or double counting peaks (that is, our algorithm often failed to find a peak, or counted two peaks at one cycle of heartbeat in the previous process due to noisy data), we had to calculate an average value that took these variations into account, instead of the mean value. To address this, we defined *M*′ for each period such that f(M′) below reached the minimum, with *M*′ ranging from 15 to 40 frames in increments of 0.01.

$$\begin{array}{c} f\left(M^{\prime }\right)=\sum_{k}\min_{a\in \left\{2,1,0.5\right\}}{\left(aPPI_{k}-M^{\prime }\right)}^{2} \end{array}$$

Subsequently, we computed the average heart rate *h* (beats per minute; identified with the pulse rate in this study) as the inverse of *M*′.

We determined the average heart rate for (a) the baseline period; (b) the first time period; and (c) the second time period. (a) The baseline period corresponds to the first 30 s of trial measurement, during which the participants were not presented with stimuli of calligraphy works. The (b) first time period and (c) second time period correspond to the first and second halves of each 3-min presentation of the calligraphy work, respectively. We cut off the first 5 s of (b) and the last 5 s of (c) to remove artifacts resulting from bandpass filtering.

After calculating the average heart rate for each period, we excluded any clearly inaccurate or doubtful data from the analysis of heart rate (for the criteria, see Supplementary Table 4). As a result, we did not include 40.00% of participants in that analysis (see Supplementary Text 1 and Supplementary Table 5 for the reliability of the analytical procedures).

### Results and Discussion

For each variable measured more than once, we calculated each participant’s score by averaging the stimuli in each session, which, in turn, we used for statistical analysis as a data point. Unless otherwise stated, we examined each dependent variable using multiple regression analysis. We included a dummy variable for the condition of instruction, the score for the frequency of viewing calligraphy, the interaction term of both variables, and (additionally in Study 2) the place of recruitment as predictor variables. Different from Study 1, we did not include scores of liking in the analysis, while this time the length of time spent learning calligraphy was included as a predictor variable in some post-hoc analyses.

#### Validation of Assignments and Experimental Manipulation

To check whether we appropriately assigned the participants in terms of their attitudes toward calligraphy, we calculated the mean and SD of their scores for the frequency of viewing calligraphy for each condition (see Table 3). As the independent *t*-test showed no significant differences among them, *t*(77) = 0.813 *p* = 0.419, there is no evidence to suggest that the assignments were biased.

**TABLE 3:** Descriptive statistics for all rating values in Study 2.

Variable	Tracing group	Control group
Frequency of viewing	0.42 (0.65)	0.30 (0.60)
Awareness of physical creation	4.36 (1.11)	3.74 (1.27)
Awareness of mental creation	3.37 (1.56)	3.09 (1.78)
Admiration	2.89 (1.07)	2.50 (1.27)
Inspiration	2.64 (1.22)	2.74 (1.45)
Empathy	2.57 (1.14)	2.33 (1.34)
Imagination	3.22 (1.10)	2.95 (1.15)

*Standard deviations appear in parentheses.*

Further, to confirm whether the manipulation of the instructions influenced the participants’ cognitive processes as expected, we calculated the mean and SD of their scores for (a) the degree to which they were aware of the physical process of writing calligraphy (awareness of physical creation) and (b) the degree to which they were aware of the mental process of writing calligraphy (awareness of mental creation; see Table 3). As for (a) awareness of physical creation, the outcomes showed that the effects were significant for the condition, *b* = 0.76, *t*(74) = 2.46, *p* = 0.016, and the frequency of viewing calligraphy, *b* = 0.76, *t*(74) = 2.55, *p* = 0.013, but the other effects were insignificant (Table 4). As for (b) awareness of mental creation, the results indicated that the effects were insignificant for all predictors (Table 4). These findings imply that the manipulation in the current study had a similar function to that of Study 1, and changed the viewer’s recognition of the writing process of calligraphy to some extent, but did not change as radically as that of Study 1, since there was no significant difference between the conditions in terms of mental creation (at least consciously). The reason why the effect was weakened (despite using the same texts regarding calligraphy) may be that the relatively complicated procedures of using a smartphone to measure PPG distracted the participants from contemplating the calligraphy. Therefore, we considered the manipulation to be validated enough to create some differences between conditions, but less effective than Study 1, perhaps making some effects unobservable.

**TABLE 4:** Multiple regression analyses in Study 2.

Dependent variable		Predictor	*b*	*t*	*p*
Awareness of physical creation					
		Intercept	3.61***	14.08	<0.001
		Condition (Tracing: 1, Control: 0)	0.76*	2.46	0.016
		Frequency of viewing	0.76*	2.55	0.013
		Interaction (condition × frequency)	−0.56	−1.31	0.194
		Place of recruitment	−0.18	−0.65	0.521
Awareness of mental creation					
		Intercept	3.04***	8.33	<0.001
		Condition (Tracing: 1, Control: 0)	0.11	0.24	0.812
		Frequency of viewing	0.63	1.48	0.143
		Interaction (condition × frequency)	0.31	0.52	0.607
		Place of recruitment	−0.24	−0.59	0.555
Admiration					
		Intercept	2.61***	10.44	<0.001
		Condition (Tracing: 1, Control: 0)	0.60*	2.01	0.048
		Frequency of viewing	0.61*	2.10	0.039
		Interaction (condition × frequency)	−0.33	−0.81	0.422
		Place of recruitment	−0.50	−1.81	0.074
Inspiration					
		Intercept	2.70***	10.12	<0.001
		Condition (Tracing: 1, Control: 0)	0.10	0.30	0.761
		Frequency of viewing	1.07***	3.44	<0.001
		Interaction (condition × frequency)	−0.39	−0.89	0.375
		Place of recruitment	−0.48	−1.63	0.106
Empathy					
		Intercept	2.50***	9.44	<0.001
		Condition (Tracing: 1, Control: 0)	0.19	0.60	0.551
		Frequency of viewing	0.50	1.64	0.106
		Interaction (condition × frequency)	0.22	0.49	0.623
		Place of recruitment	−0.54	−1.86	0.067
Imagination					
		Intercept	2.92***	11.86	<0.001
		Condition (Tracing: 1, Control: 0)	0.33	1.11	0.271
		Frequency of viewing	0.53	1.86	0.067
		Interaction (condition × frequency)	−0.15	−0.37	0.713
		Place of recruitment	−0.24	−0.87	0.386
Heart rate change ratio for first time period: log (*h*_p1_ / *h*_b_)					
		Intercept	0.00	0.18	0.859
		Condition (Tracing: 1, Control: 0)	−0.01	−0.54	0.592
		Frequency of viewing	−0.03	−1.06	0.297
		Interaction (condition × frequency)	0.05	1.33	0.191
		Place of recruitment	0.01	0.51	0.610
Heart rate change ratio for second time period: log (*h*_p2_ / *h*_b_)					
		Intercept	0.00	0.20	0.844
		Condition (Tracing: 1, Control: 0)	−0.02	−0.67	0.505
		Frequency of viewing	−0.05*	−2.13	0.039
		Interaction (condition × frequency)	0.08*	2.28	0.028
		Place of recruitment	0.01	0.59	0.559
	(*Post hoc* analysis for low-frequency group)				
		Intercept	0.02	0.92	0.366
		Condition (Tracing: 1, Control: 0)	−0.05*	−2.08	0.047
		Time spent learning calligraphy	−0.05*	−2.91	0.007
		Interaction (condition × time)	0.08	1.93	0.064
		Place of recruitment	0.02	1.04	0.310
	(*Post hoc* analysis for high-frequency group)				
		Intercept	0.03	0.58	0.573
		Condition (Tracing: 1, Control: 0)	0.07	1.05	0.320
		Time spent learning calligraphy	−0.05	−2.15	0.057
		Interaction (condition × time)	0.00	0.00	0.998
		Place of recruitment	−0.00	−0.07	0.949

*p < 09.05; **p < 0.01; ***p < 0.001.

#### Aesthetic Impression

We calculated the mean and SD of the participants’ scores for the admiration of calligraphy (Table 3 and Figure 3). Multiple regression analysis only revealed significant effects for the condition, *t*(74) = 2.01, *p* = 0.048 and the frequency of viewing, *t*(74) = 2.10, *p* = 0.039 (Table 4).

This outcome is largely consistent with our hypothesis and the result in Study 1 in terms of supporting the idea that admiration is elicited by the change in recognition of the writing process of calligraphy. Although there is another significant effect of the frequency of viewing, since that effect in Study 1 was also near the significant level (*p* = 0.093), there seems to be no essential difference between Study 1 and 2. In addition, because expertise often has a positive impact on the viewer’s aesthetic impression (cf. Leder et al., 2012; van Paasschen et al., 2015; Matsumoto and Okada, 2019), the fact that the frequency of viewing positively correlates with admiration is not surprising.

#### Inspiration and Other Rating Items

We calculated the mean and SD of participants’ scores on inspiration by viewing calligraphy (Cronbach’s alpha coefficient was 0.84; Table 3). Multiple regression analysis only revealed a significant effect for the frequency of viewing, *t*(74) = 3.44, *p* < 0.001 (Table 4). Likewise, we calculated the mean and SD of participants’ scores for empathy and imagination (Table 3). For both variables, multiple regression analysis showed no significant effect for all predictors (Table 4).

Based on these results, we can conclude that the manipulation of instructions and orientation of cognitive processes for viewing calligraphy had no observable effects other than admiration, the construct most closely related to the recognition of the writing process of calligraphy among all items in Study 2. The other effects of the tracing method suggested by Study 1 were not shown in Study 2; this seems to be because they were diluted by the complicated procedures of measuring PPG, as well as the index of mental creation described in “Validation of assignments and experimental manipulation.” In addition, any psychological variables used in this study except inspiration are measured by single-item scales, and their reliability cannot be assessed by Cronbach’s alpha coefficient but may possibly be low. This lack of reliability may potentially account for the difference between the two studies. Therefore, although the effects on inspiration and empathy observed in Study 1 are not so robust (at least as much as admiration), and the effects on imagination suggested in Study 1 are not supported either, whether or not these variables are influenced by the way one recognizes the writing process of calligraphy should not be determined based on the current findings alone.

#### Heart Rate

**FIGURE 7:**
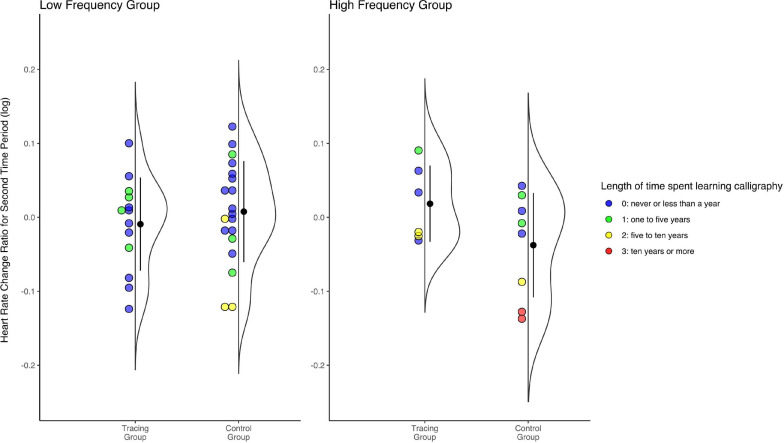
Plot showing distributions of heart rate change ratio for the second time period in both groups of low frequency **(left)** and high frequency **(right)** of viewing calligraphy. Each dot represents individual values (color-coded by the length of time spent learning calligraphy). Violin plots to the right of the colored dot plots depict distributions of corresponding data, using the “geom_flat_violin” function (Robinson, 2015). Black dots and bars in violin plots represent each mean and standard deviation.

We calculated the mean and SD of the participants’ average heart rate from PPG signals for the baseline (*h*_b_), the first time period (*h*_p1_), and the second time period (*h*_p2_). In the Tracing Group, the mean (SD) of *h*_b_, *h*_p1_, and *h*_p2_ was 81.52 (10.50), 82.37 (13.10), and 82.44 (12.41) beats per minute, respectively. In the Control Group, this was 79.27 (8.47), 79.40 (8.24), and 78.73 (7.31) beats per minute, respectively. Subsequently, we calculated the ratio of *h*_p1_ and *h*_p2_ to *h*_b_ for each participant. We used the natural logarithmic value of this ratio as the response variable in the multiple regression analysis, with the same predictor variables as other psychological measurements. While there was no significant effect for all predictors for the first period, for the second time period, the effect were significant for the frequency of viewing, *b* = −0.05, *t*(42) = −2.13, *p* = 0.039 and the interaction, *b* = 0.08, *t*(42) = 2.28, *p* = 0.028, but the other effects were insignificant (Table 4). To examine the nature of the interaction effect via post-hoc analysis, we further divided the participants into two groups based on their scores for the frequency of viewing (the low-frequency group of participants scoring 0: those who “almost never” have the opportunity to appreciate works of calligraphy, *n*_t_ = 13, *n*_c_ = 19; and the high-frequency group of participants scoring 1 or greater: those who have the opportunity to appreciate works of calligraphy “once every few years” or more, *n*_t_ = 7, *n*_c_ = 8; see also Supplementary Table 1). We carried out multiple regression analysis for each group with four predictor variables (length of time spent learning calligraphy and its interaction term with condition were added to condition and place of recruitment) and the same dependent variable. While the condition had a significant effect in the low-frequency group, *b* = −0.05, *t*(27) = −2.08, *p* = 0.047, it had no significant effect in the high-frequency group, *b* = 0.07, *t*(10) = 1.05, *p* = 0.320 (Figure 7). In addition, there was significant effect of length of time spent learning calligraphy only in the low-frequency group, *b* = −0.05, *t*(27) = −2.91, *p* = 0.007. The effect of place of recruitment was insignificant for both groups (Table 4). This indicates that the influence of the condition in all samples was especially derived from differences among participants who did not have the habit of appreciating calligraphy.

If we focus on the simple main effect for the low-frequency group, the current result can be interpreted in a way that is straightforwardly consistent with previous studies. The extant literature suggests that heart rate during task execution is positively correlated with boredom (London et al., 1972; Merrifield and Danckert, 2014; Raffaelli et al., 2018). This trend also applies to the presentation of aesthetic objects; that is, attention to (and interest in) stimuli that are associated with lower heart rate responses (Libby et al., 1973). Therefore, the currently observed effect of the changing ratio of heart rate between conditions may mean that the participants in the Tracing Group remained interested in the art without becoming bored, which resulted from the tracing method. Additionally, there was also the effect of time spent learning calligraphy, which affected the low-frequency group in the same way that the tracing method did. That is, the longer a participant spent learning calligraphy, the lower the participant’s heart rate, which resulted from the participant’s maintaining interest in the stimuli. To put it in another way, the current results of the PPG suggest that, in terms of how calligraphy is viewed, the tracing method may, to some extent, substitute for the effects obtained by the long-term study of calligraphy. We should also note that condition had no effect during the first time period. One possibility is that participants in both the Tracing Group and Control Group viewed works without boredom in the beginning, but those in the Control Group gradually became bored because they lacked of an appropriate strategy for viewing the art, whereas those in the Tracing Group remained interested.

There are likely a few reasons why the results concerning the difference in change in heart rate ratio between conditions differed between the low-frequency and high-frequency groups. First, there were not enough participants in the high-frequency group (because the majority of participants had seldom engaged in calligraphy appreciation), which may be the main reason why the post-hoc analysis in the high-frequency group did not yield any significant effect. Second, there is the possibility that, especially for people who are accustomed to regular calligraphy appreciation, introducing a new technique such as the tracing method has a neutral or even obstructive effect. The tracing method might interfere with the viewer’s usual behavior patterns, which may have been established by frequent exposure to calligraphy. Even though the lack of significance for the high-frequency group in the post-hoc analysis may owe to the small sample size, based on the above view concerning the interaction between frequency of appreciation and experimental intervention, it is also possible that even with more participants in the high-frequency group, the heart rate of the Tracing Group might be nearly the same or even higher (rather than lower) than that of the Control Group because of the decreased interest of experienced participants.

By reflecting on the current analysis of PPG, we can see that the smartphone-based PPG can be used effectively in a Web experiment. At the same time, however, there are limitations regarding the accuracy of this measurement. The fact that we could not use about 40% of the data of all participants for the analysis of heart rate, due to various noises, clearly shows the magnitude of this problem. Hence, future research should refine the smartphone-based PPG method and involve follow-up testing of the results using more reliable equipment.

## General Discussion

We will summarize the findings of Studies 1 and 2 as follows (see Table 5 for a comparison of results). By orientating viewers’ cognition about the recognition of the process of creating artworks through manipulated instructions about how to perceive the art (i.e., suggesting the “tracing method,” the procedure of viewing calligraphy in the order in which lines were drawn as if by “tracing” with one’s eyes), viewers will have deeper admiration. This outcome is not only consistent with both of the experiments, but also supports the findings of Matsumoto and Okada (2019), whereby detailed recognition of the process of creating artworks leads to admiration. As for inspiration and empathy, Study 1 showed significant effects of the tracing method, while Study 2 did not. Since it is likely that the complicated procedures of Study 2 distracted the participants from appreciating the calligraphy, the relationship between those variables and recognition of the process of creating artworks requires further investigation. Study 2 further probed viewers’ physiological responses related to the autonomic nervous system by smartphone-based PPG. As a result – especially for participants who view calligraphy relatively less frequently – we found a significant difference between conditions in the ratio of the average heart rate in the second half of the duration of viewing stimuli compared to the baseline, with lower ratio for those who were given tracing method. This suggests that the tracing method may allow viewers to continue contemplating calligraphy without growing bored, even during the latter half of a fairly long viewing time (although this is only one possibility).

**TABLE 5:** Comparison of results of Likert scales in Studies 1 and 2.

Variable	Study 1	Study 2
Frequency of viewing	n.s.	n.s.
Awareness of physical creation	Condition	Condition frequency of viewing
Awareness of mental creation	Condition frequency of viewing	n.s.
Admiration	Condition	Condition frequency of viewing
Liking of a work	n.s.	(Not included in analysis)
Inspiration	Condition frequency of viewing	Frequency of viewing
Empathy	Condition	n.s.
Imagination	Frequency of viewing	n.s.

*n.s. = not significant for all predictor variables. In cases where there is any significant effect in each result of regression analysis, only significant predictor variables are listed. See Tables 1–4 and Figures 3, 7 for details of each result.*

This study contributes to the literature on the psychology of aesthetics in the following ways. First, like previous research (Matsumoto and Okada, 2019), this study demonstrates that the role played in the art-viewing process by recognition of the process of creating artworks – which has not been highlighted in the dominant models of art-viewing (e.g., Leder et al., 2004; Pelowski et al., 2017) – is large and robust in both studies. Our investigation into recognition of the process of creating artworks was made possible in large part by the characteristics of Chinese/Japanese calligraphy, as they enable the method of viewing by tracing, which is hard to apply directly to other kinds of art (such as painting). This does not mean that application of our findings must be limited to the field of calligraphy, because the mental mechanism of feeling admiration – imagining the process of another person creating an artwork, making a social comparison between oneself and others, and then feeling admiration – can be assumed in any kind of art-viewing (Matsumoto and Okada, 2019). Rather, this study is an example of how psychological research on the arts in relatively atypical artistic domains can provide deeper insight into vital aspects of the cognitive process of art-viewing, which have yet to be adequately addressed. Second, this study revealed the inspirational process caused by viewing art. The way in which art appreciation leads to inspiration has rarely been dealt with in experimentally controlled situations; our study is also valuable in that regard (see Ishiguro and Okada, 2019, for an example of a questionnaire survey). Up until now, in virtually all models of art appreciation, interest has been exclusively focused on the range of time between viewers’ initial perceptions of art and their impression formation. In the current study, we made a first step to empirically explore the mental process, starting from art appreciation and expanding to other activities of viewers. Third, from a practical perspective, this study has implications for how to display art to allow viewers to contemplate it in such a way that even novices can deeply appreciate it. Although recognition of the process of creating artworks has an important role in art-viewing, the results of Study 1 imply that simply thinking about that process while looking at them will not make any notable difference. On the other hand, at least for calligraphy, we can expect that an instruction as simple as the “tracing method” will satisfy viewers to some degree, even in a practical situation. Since this is not only effective but also simple and short, our findings are practically significant for art education or museum management. Moreover, it is quite possible that as with the tracing method in calligraphy, guidance orientating viewers toward a detailed recognition of the process of creation is generally effective for viewing other types of art. Specific methods can be addressed in future research.

Further, this study has significance in that it introduces a new perspective to graphonomics. By applying the theory of the psychology of aesthetics and conducting experiments, we clarified a part of the process of forming aesthetic impressions (especially admiration) of other people’s handwriting, which remains almost unexamined in graphonomics. Since admiration and inspiration are classified as assimilative emotions rather than contrastive ones (Smith, 2000), when people aim to write well, they might tend to model themselves after the handwriting of others whom they admire, whether consciously or not. Therefore, starting from this study and performing more detailed analysis to find out how the physical characteristics of handwriting are associated with the formation of mental representations about the creative process, it may become clearer what kinds of handwriting we admire, and what we internally represent as a goal when writing. This will have important implications for graphonomic research, which addresses the process of human writing. The possibility that written language can have “paralinguistic” cues and communicate information about the writer has not received much attention in recent graphonomic research. This may be due to the longstanding criticism of graphology that the image of the writer that the reader constructs, using the letters as clues, does not reflect reality (King and Koehler, 2000; Simner and Goffin, 2003). However, this study suggests that – especially in the art of calligraphy – the information about the author conveyed by the characters is closely linked to aesthetic feelings. Hence, by introducing a communicative perspective, future graphonomic studies are likely to reveal new aspects of human activities linked to written language. At the same time, this study shows the communicative aspect of art-related activities, which has been drawing increasing attention (e.g., Dolese, 2015). Our findings span the two subfields of communication research – written language and art – and should contribute to the holistic understanding of human communication.

This study is also very valuable as a practical example of measuring a physiological parameter, without any specialized equipment, in a remote environment. The outcome of average heart rate in this study is consistent with existing findings. In this respect, our study supports the usefulness of smartphone-based PPG in empirical aesthetics or other kinds of psycho-physiological research. This will be especially effective when a researcher cannot conduct face-to-face experiments with participants or examine their daily activities in vivo. On the other hand, smartphone-based PPGs tend to be noisy and require caution in their use in situations without face-to-face supervision by an experimenter. Future research should consider elements such as the type of device suitable for measurement and the wording of instructions to ensure that participants can perform the measurement accurately.

Finally, future research could go in several directions: (1) examining different forms of characters and letters from the ones dealt with in this study, namely, handwriting in ordinary situations or printed fonts; (2) confirming whether presenting the actual process of creating artworks through media (such as video) will have a similar effect to that observed in this study; (3) adding a rating scale of boredom and examining correlations between it and heart rate to verify the current discussion on heart rate; (4) measuring other indices (such as skin conductance or eye movements) for a more multifaceted understanding of art appreciation and impression formation for letters.

## Data Availability Statement

The datasets presented in this study can be found in online repositories. The names of the repository/repositories and accession number(s) can be found below: https://github.com/psychologyKM/2020experiments.

## Ethics Statement

Ethical review and approval was not required for the study on human participants in accordance with the local legislation and institutional requirements. Written informed consent for participation was not required for this study in accordance with the national legislation and the institutional requirements.

## Author Contributions

KM designed and performed the experiments, analyzed the data, and co-wrote the manuscript. TO supervised the research and co-wrote the manuscript.

## Conflict of Interest

The authors declare that the research was conducted in the absence of any commercial or financial relationships that could be construed as a potential conflict of interest.
