# Supplementary material for: Imagining How Lines Were Drawn: The Appreciation of Calligraphy and the Facilitative Factor Based on the Viewer’s Rating and Heart Rate
Source: Front Hum Neurosci. 2021 Jun 30;15:654610. doi: 10.3389/fnhum.2021.654610 (PMC8279771; doi:10.3389/fnhum.2021.654610)
Supplement: Supplementary file 1 [file Table_1.DOCX]

Supplementary Material

Supplemental Text S1
*Detailed Process of Verifying the Reliability of the Smartphone-based PPG.*

We obtained data from two participants (26 and 34-year old men, respectively). After one or two trials, they measured their pulse rate over a three-minute period by the following two means: (1) the PPG method, which is same as that used in Study 2; and (2) a smartwatch with the ability to measure pulse rate (used as the “gold standard” to assess the reliability of (1)). While PPG in (1) was obtained from the tip of the index finger of the left hand, the smartwatch in (2) created the PPG signal from the skin of the left wrist. The iPad Pro (10.5 inches, 2nd generation) and Apple Watch (40mm, Series 6) were used in (1) and (2), respectively.

In the analysis, we employed all data on heart rate provided by the smartwatch as a form of time series data, with a data point roughly every few seconds. For every timing when each data point was obtained by the smartwatch’s default algorithm, the pulse rates were calculated based on the average pulse-to-pulse intervals obtained from the PPG signal in (1) for 10 seconds before and after those timings. In the end, we computed 29 or 28 data points for comparison for each participant.

We calculated the mean and SDs of differences in estimated heart rate (beats per minute) between (1) and (2) for each data point and each participant. The results are shown in Supplemental Table S3. According to the outcomes, there was only an average difference of less than 1 bpm between the estimation based on (1) and (2) when averaged over the whole measurement duration. Therefore, the procedure for measuring PPG employed in Study 2 is sufficiently accurate to be of practical use.

Table S1
*Scales adopted in Studies 1 and 2 (except those discarded in the analyses)*

| Variable | Text | Answer options |
| --- | --- | --- |
| Frequency of viewing | How often do you appreciate works of calligraphy?  あなたは書道の作品を鑑賞することがどの程度の頻度でありますか？ | 4-point scale (3: “every month or more,” 2: “more than every year and less than every month,” 1: “more than once every few years and less than every year,” 0: “almost never”) |
| Length of time spent learning calligraphy | How much experience do you have in learning about calligraphy? (Cumulative number of years, from which experiences that may have been shared by many people around you, such as classes at elementary and junior high schools should be excluded)  あなたは書道に関して学んだ経験をどれくらい持っていますか？累計の年数でお答えください。ただし、小中学校での一斉授業など、あなたの周りの多くの人が共有していたと考えられる経験は除外して、書道のための教室や自主的に学んだ時間などのみ計算に入れてください。 | 4-point scale (3: “ten years or more,” 2: “five to ten years,” 1: “one to five years,” 0: “never or less than a year”) |
| Awareness of Physical Creation* | I was aware of how the brush was moved.  どのように筆が動かされたかに意識を向けた | 7-point scale from “Agree very strongly” (6) to “Disagree very strongly” (0) |
| Awareness of Mental Creation* | I was aware of what calligrapher thought.  書き手が何を考えたかに意識を向けた |  |
| Admiration* | I felt admiration.  感嘆した |  |
| Liking of a work* | I like this work.  この作品が好きである |  |
| Liking of meaning of word* | I like the content of the word written here.  ここに書かれている言葉の中身が好きである |  |
| Empathy* | I empathized with the calligrapher.  書き手に共感した |  |
| Imagination* | My imagination expanded from the work.  作品から想像が広がった |  |
| Inspiration | I felt inspired. インスピレーション（触発）を感じた |  |
|  | New images and ideas came to mind.  新しいイメージやアイディアが湧いた |  |
|  | I was excited.  わくわくした |  |
|  | I wanted to express myself in some way. 自分も何か表現したくなった |  |
|  | I wanted to actually do something.  実際に何かをしてみたくなった |  |

*Note.* The items marked with an asterisk were presented in as many repetitions as the number of stimuli (i.e., four times). Although inspiration was originally measured separately for intensity and frequency (Ishiguro & Okada, 2015; Thrash & Elliot, 2003) with options such as “never,” “very often,” “not at all,” or “very strongly,” we only measured its intensity with options about agreement for the following reasons: 1) A shortened scale will reduce the burden on the participants. 2) In the current context, frequency and intensity seem almost indistinguishable for participants. 3) When the answer options are same as other items, participants are less likely to make mistakes in their responses.

Table S2
*Information about each work.*

| Presenting order | Image | Description |
| --- | --- | --- |
| 1 | 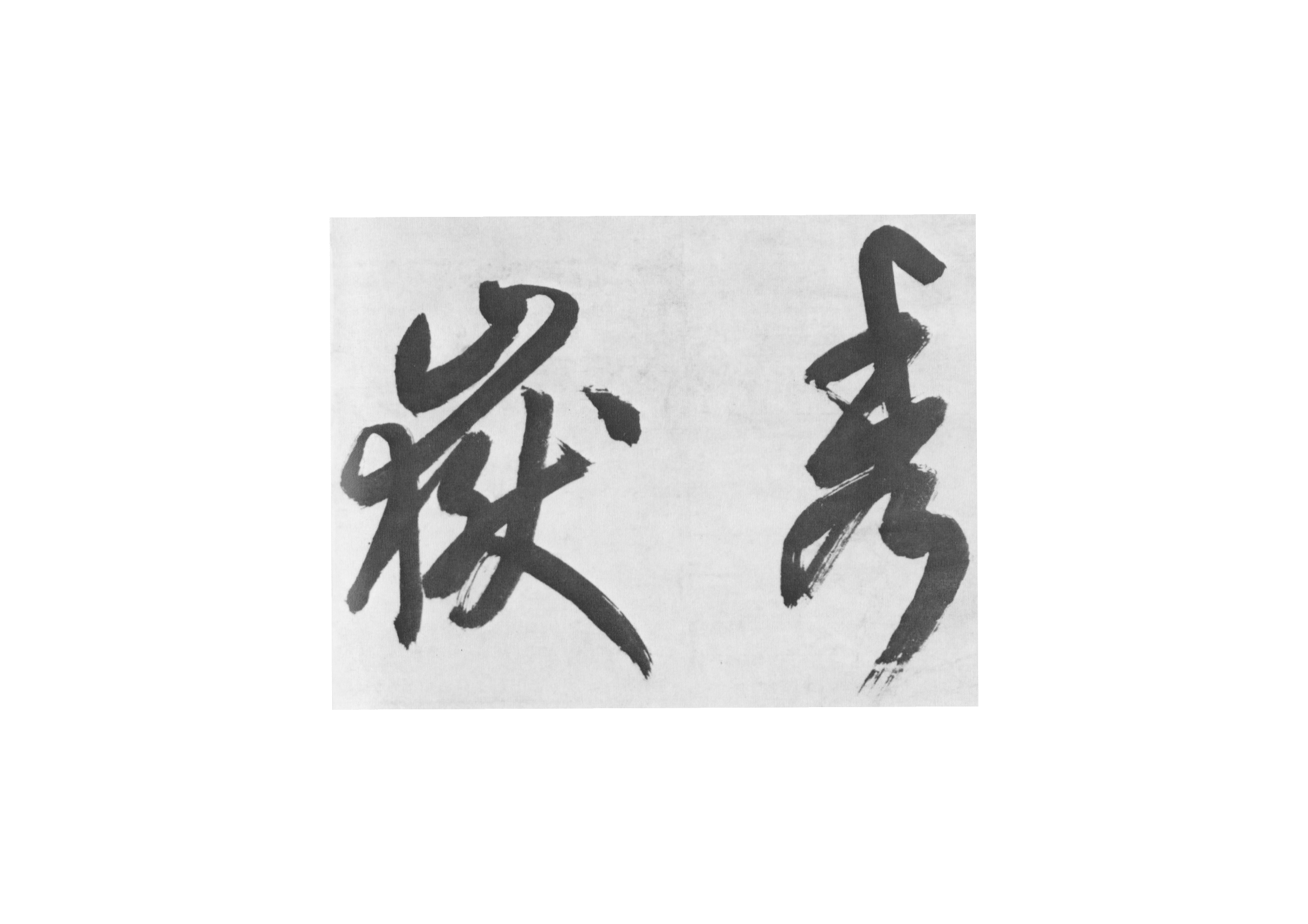 | **Written word**: Shu-Gaku (秀嶽)*  **Meaning of word**: A mountain that is much higher and larger than others.  **Calligrapher**: Takuan (沢庵, 1573 - 1646)  *read from right to left |
| 2 | 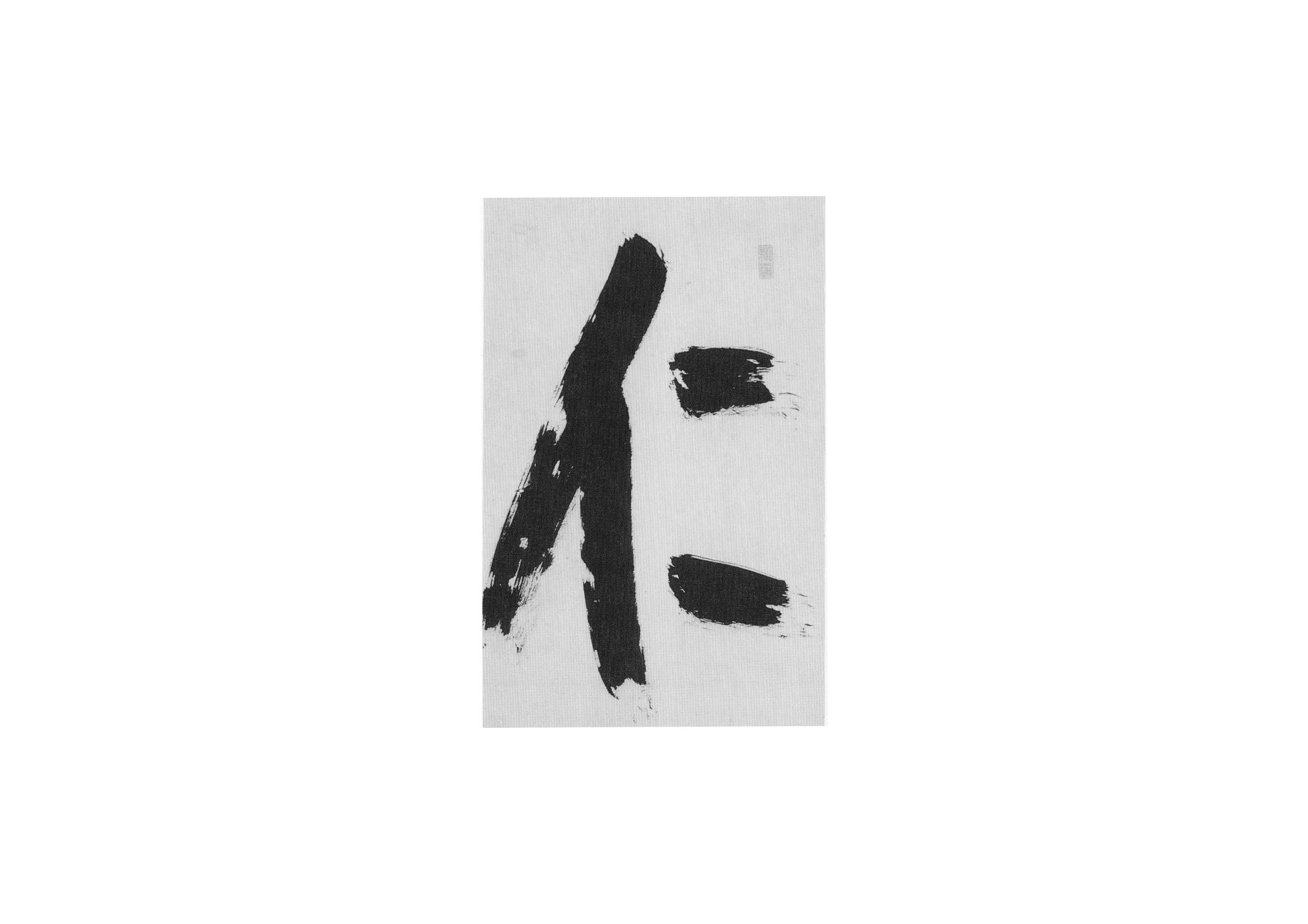 | **Written word**: Jin (仁)  **Meaning of word**: Compassion. Kindness. Mercy.  **Calligrapher**: Jiun-Sonja (慈雲尊者, 1718 - 1804) |
| 3 | 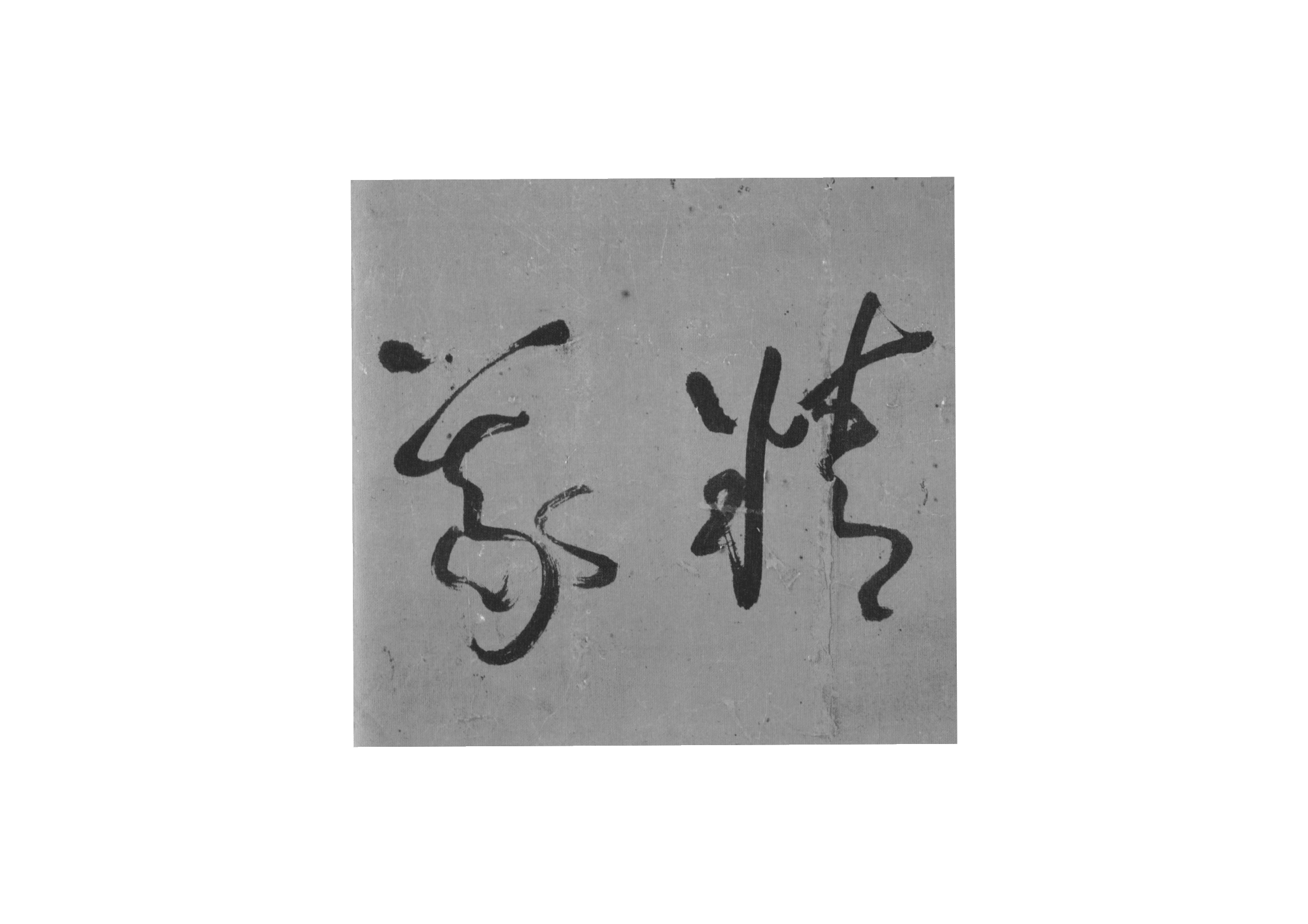 | **Written word**: Sei-Gi (精義)*  **Meaning of word**: Exact significance. Detailed interpretation.  **Calligrapher**: Kameda-Bōsai (亀田鵬斎, 1752 - 1826)  *read from right to left |
| 4 | 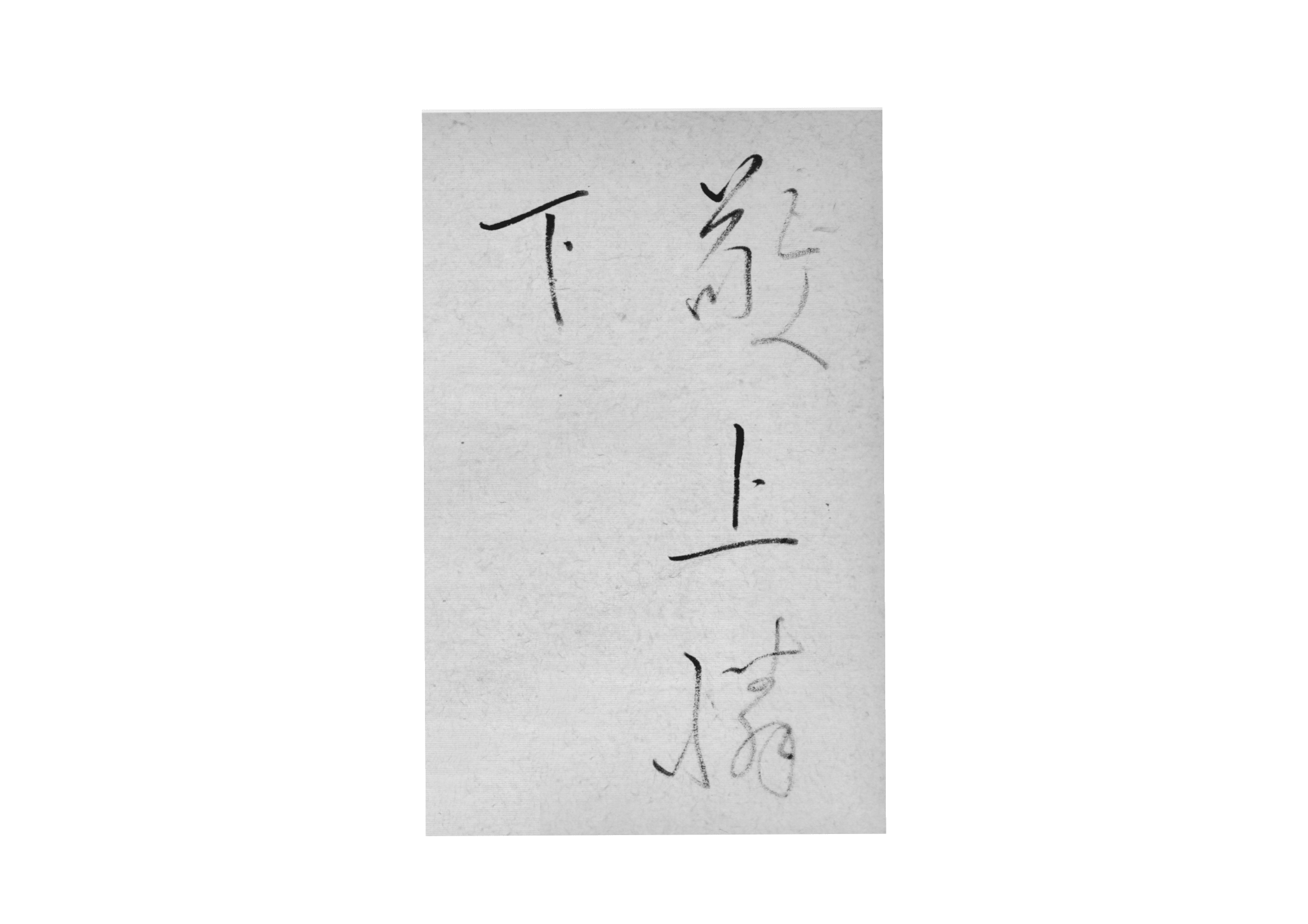 | **Written word**: Kei-Jō-Ren-Ge (敬上憐下)*  **Meaning of word**: To respect those who are higher in rank or position, and to have compassion fpr those who are lower in them.  **Calligrapher**: Ryōkan (良寛, 1758 - 1831)  *read from up to down and right to left |

Table S3  *Instructions foreach condition.*

| Condition | Instruction |
| --- | --- |
| Tracing Group | - Please view a work with the intention of following strokes one by one, as if tracing the path of the brush with your eyes. - When viewing calligraphy, it is essential to "read brush idea”. This means that it is important to use your imagination to read with imagining what kind of intentions and feelings were put into the brush strokes and the characters themselves. - By tracing the brushstrokes with your eyes and replaying in your mind the process of them as they are written, it becomes easier for even beginners to “read brush idea.” - Of course, there are no prohibitions at all, such as "don't look at it this way" or "don't think this way.” Basically, you are free to view the works in any way you like. |
| No-Tracing Group | - Please view a work with the imagination of the process by which the work was created. - When viewing calligraphy, it is essential to "read brush idea”. This means that it is important to use your imagination to read with imagining what kind of intentions and feelings were put into the brush strokes and the characters themselves. - By consciously imagine the background of the work, it becomes easier for even beginners to “read brush idea.” - Of course, there are no prohibitions at all, such as "don't look at it this way" or "don't think this way.” Basically, you are free to view the works in any way you like. |
| Control Group | - You are free to think or imagine whatever you like while looking at the work. - Of course, there are no prohibitions at all, such as "don't look at it this way" or "don't think this way.” Basically, you are free to view the works in any way you like. |

*Note*. The parts that are described differently between Tracing Group and No-Tracing Group are marked with a solid underline. The parts where the description is exactly the same between all groups are marked with a broken underline.

Table S4
*A summary of data-exclusion in estimating heart rates.*

| Criteria | Number of participants | | |
| --- | --- | --- | --- |
|  | Before removal | Removed | After removal |
| 1. We removed files with inadequate electronic sound recording or recording period. | 80 | 2 | 78 |
| 1. After the calculation of *M’*, we removed every data point for which *M’* was calculated to be 15.00 or 40.00, because it indicates that *M'* most likely did not have a local solution between 15 and 40, the predetermined range. | 78 | 0 | 0 |
| 1. We removed participants for whom baseline data had already been removed or for whom there was less than two data sets that can be accepted for both the first and second periods. Furthermore, for each participant, SD of *M’* s from remaining data (containing baseline, first and second periods) was obtained and if SD > 2.00, that participant was wholly excluded because of excessive variability in heart rate estimates (indicating that the estimates were likely to be inaccurate). | 78 | 30 | 48 |

| Participant | Mean (SD) of heart rate (smartphone) [BPM] | Mean (SD) of heart rate (gold standard) [BPM] | Mean (SD) of differences | Mean absolute deviation |
| --- | --- | --- | --- | --- |
| ID:1 (age = 26) | 86.27 (2.82) | 86.46 (1.57) | -0.19 (3.30) | 2.39 |
| ID:2 (age = 34) | 75.31 (2.31) | 75.66 (2.70) | -0.34 (2.29) | 1.85 |

Table S5
*Comparison of two measurements for the reliability verification.*
